# The *Drosophila melanogaster* Phospholipid Flippase dATP8B Is Required for Odorant Receptor Function

**DOI:** 10.1371/journal.pgen.1004209

**Published:** 2014-03-20

**Authors:** Yu-Chi Liu, Michelle W. Pearce, Takahiro Honda, Travis K. Johnson, Sandhya Charlu, Kavita R. Sharma, Mays Imad, Richard E. Burke, Konrad E. Zinsmaier, Anandasankar Ray, Anupama Dahanukar, Marien de Bruyne, Coral G. Warr

**Affiliations:** 1 School of Biological Sciences, Monash University, Clayton, Victoria, Australia; 2 Department of Biochemistry and Molecular Biology, Monash University, Clayton, Victoria, Australia; 3 Department of Entomology, University of California, Riverside, California, United States of America; 4 Department of Neuroscience, University of Arizona, Tucson, Arizona, United States of America; 5 Department of Molecular & Cellular Biology, University of Arizona, Tucson, Arizona, United States of America; North Carolina State University, United States of America

## Abstract

The olfactory systems of insects are fundamental to all aspects of their behaviour, and insect olfactory receptor neurons (ORNs) exhibit exquisite specificity and sensitivity to a wide range of environmental cues. In *Drosophila melanogaster*, ORN responses are determined by three different receptor families, the odorant (Or), ionotropic-like (IR) and gustatory (Gr) receptors. However, the precise mechanisms of signalling by these different receptor families are not fully understood. Here we report the unexpected finding that the type 4 P-type ATPase phospholipid transporter *dATP8B*, the homologue of a protein associated with intrahepatic cholestasis and hearing loss in humans, is crucial for *Drosophila* olfactory responses. Mutations in *dATP8B* severely attenuate sensitivity of odorant detection specifically in Or-expressing ORNs, but do not affect responses mediated by IR or Gr receptors. Accordingly, we find *dATP8B* to be expressed in ORNs and localised to the dendritic membrane of the olfactory neurons where signal transduction occurs. Localisation of Or proteins to the dendrites is unaffected in *dATP8B* mutants, as is dendrite morphology, suggesting instead that dATP8B is critical for Or signalling. As dATP8B is a member of the phospholipid flippase family of ATPases, which function to determine asymmetry in phospholipid composition between the outer and inner leaflets of plasma membranes, our findings suggest a requirement for phospholipid asymmetry in the signalling of a specific family of chemoreceptor proteins.

## Author Summary

The olfactory systems of insects are fundamental to critical behaviours such as finding mates, food and host plants. Insects can detect a wide range of environmental cues using three different families of olfactory receptor proteins. Why insects have three different families of receptor genes, and how they function together, is not fully understood. Here we identified a new gene, *dATP8B*, which is critically and specifically required for the function of only one of these receptor families in *Drosophila*. *dATP8B* is a member of the P4-type ATPases, or phospholipid flippases; these enzymes function in establishing a difference or asymmetry in lipid composition between the outer and inner leaflets of plasma membranes. This is thought to be important for many cellular membrane processes; however, specific functions of individual flippase proteins are not well described. We find that *dATP8B* is required for the function of the odorant receptor family, but not the ionotropic-like and gustatory receptor families. This further highlights the functional differences between these receptor families and suggests a role for phospholipids in the signalling of a specific family of receptor proteins.

## Introduction

In insects such as *Drosophila melanogaster* the detection of environmental odours is achieved by arrays of olfactory receptor neurons (ORNs) housed in different types of chemosensory hairs (sensilla) on two olfactory organs, the antenna and the maxillary palp. Each class of ORN is tuned to specific chemical signals by expression of different olfactory receptor genes. The responses of most insect ORNs are reliant on members of two large and divergent families of olfactory receptor proteins, the odorant receptor (Or) and Ionotropic glutamate-like receptor (IR) families. Or proteins are seven trans-membrane domain proteins that are topologically inverted in comparison to G-protein-coupled receptors [1], [2], raising the question as to whether they do interact with G proteins. Indeed, several studies have concluded that Or-signalling is rather ionotropic, and that the functional receptor is a ligand-gated cation channel composed of a variable Or odorant-binding subunit and a co-receptor subunit called Orco [1]–[4]. Orco is required for Or proteins to be transported to the dendrites [5], and heterologous expression studies suggest Orco is also part of the functional receptor and is essential for the initial fast inward current upon ligand binding [6], [7]. However, there is also genetic and pharmacological evidence for a slower metabotropic transduction cascade (for an excellent recent review see [8]). For example, heterologous expression experiments demonstrated a slower metabotropic current after Or stimulation, as well as an increase in cAMP [4]. Orco has been suggested to be activated by cAMP [4], and in addition it has been suggested that phosphorylation of Orco by protein kinase C (PKC) is required for its activation [9]. However, several studies of loss of function of Gα–encoding genes in *Drosophila* have yielded conflicting results [10]–[12]. A recent study suggests that metabotropic regulation of Orco regulates Or sensitivity [13]. Overall, the mechanism of Or signalling appears to be complex, with both ionotropic and metabotropic pathway involvement, and despite much investigation is not fully understood.

*IR* genes encode a very different family of receptors, they are three trans-membrane domain ligand-gated ion channels that are related to ionotropic glutamate receptors [14]. IR proteins form heteromers but are not reliant on Orco [15], [16]. A third family of receptors, the gustatory receptors (Grs), are also seven trans-membrane proteins and are evolutionarily related to the Ors [17], [18]. Where the Or and IR families both detect a range of structurally diverse odorants and function in many ORNs [19], [20], only one functional class of ORN, specialized for detection of carbon dioxide (CO_2_), expresses *Gr* genes [21], [22]. Most other *Gr* genes are expressed in chemosensory neurons in taste sensilla on appendages of the fly that detect non-volatiles such as sugars and alkaloids [23], [24]. Although the *Grs* are evolutionarily related to *Or* genes [18], they are not reliant on Orco for function [5]. Their signalling properties have been much less extensively studied and it is not clear whether they utilize similar signalling mechanisms to Ors.

Relatively few other genes involved in the function of the peripheral olfactory system have been identified. Accordingly, to identify novel genes important for *Drosophila* peripheral olfaction, we conducted a screen for mutants defective in ORN responses. We identified a recessive mutation that specifically affects *Or*-expressing ORNs, dramatically reducing their sensitivity to ligands, but has no effect on *IR* or *Gr-*expressing sensory neurons. The causative gene, *dATP8B*, is a member of the phospholipid flippase family of P4-ATPases, which function to maintain the asymmetry in natural lipid composition between the outer and inner leaflets of cell membranes. Our results demonstrate that *dATP8B* is critically and specifically required for the function of the *Or* receptor family.

## Results

**Figure 1:**
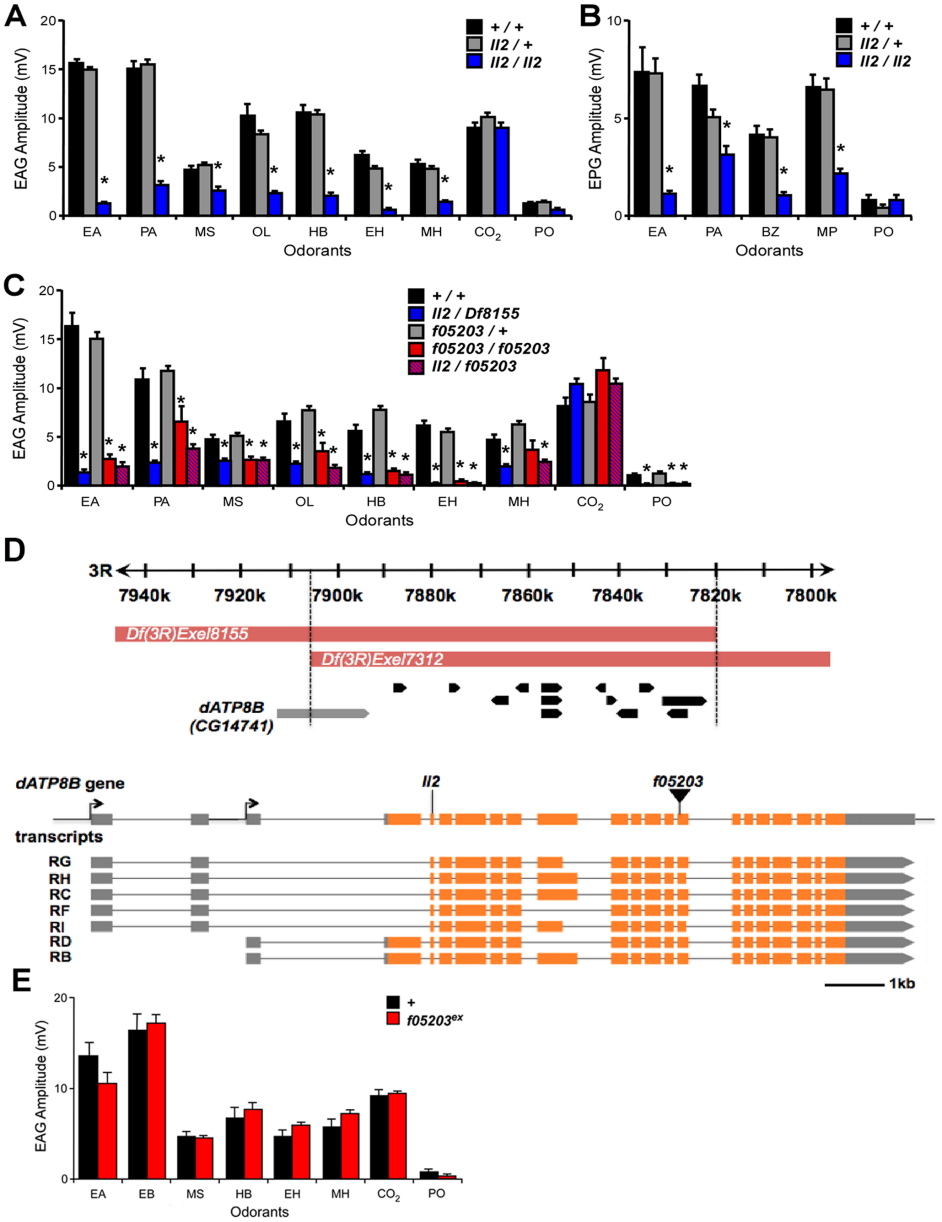
Mutations in *dATP8B* cause severe olfactory defects. (A) Electroantennogram (EAG) and (B) electropalpogram (EPG) responses to a panel of odorants. Bars represent mean response ± SEM (n = 10), asterisks are significant differences. Responses of homozygous *ll2* mutant flies (blue bars) are severely reduced when compared with controls (black bars) for all the tested odorants except CO_2_ (t-test, Bonferroni, *p<0.05*). Heterozygote flies (grey bars) are not affected. (C) A deletion that removes *dATP8B* and a *piggyBac* element inserted in *dATP8B* both fail to complement the *ll2* phenotype. Bars represent mean EAG responses ± SEM (n = 6–10). Trans-heterozygotes for *ll2* and deficiency *Df(3R)Exel8155* (blue bars) have reduced EAG response compared to controls (black bars, t-tests, Bonferroni, p<0.05), and homozygotes for the *piggyBac* insertion *dATP8B^f05203^* (red bars) and trans-heterozygotes for *ll2* and *dATP8B^f05203^* (red/blue hatch) show similar reductions when compared to heterozygote controls (grey bars; t-tests, Bonferroni, p<0.05). (D) The mapped genomic interval for the *ll2* mutant and the gene model of *dATP8B (CG14741)*. The candidate region contained 14 annotated genes. The identified nonsense mutation in *dATP8B* and the insertion site of the *piggyBac* line *dATP8B^f05203^* are in the 1^st^ and the 10^th^ common coding exon respectively, affecting all the annotated transcripts. For isoforms RC, RF, RG, RH and RI the EMS mutation causes R18-X and for isoforms RB and RD the mutation causes R197-X. Coding exons are colored in orange and the 3′ and the 5′ UTR are in grey. (E) The olfactory defect in the *dATP8B^f05203^* line is reverted when the *piggyBac* insertion is precisely excised. EAG responses of homozygous *dATP8B^f05203-Ex^* flies (red bars) to a panel of odorants were not different from controls (black bars). Bars represent mean response ± SEM (n = 5, t-test, Bonferroni). Odorants are: EA, ethyl acetate, PA, pentyl acetate, MS, methyl salicylate, OL, 1-octen-3-ol, HB, ethyl 3-hydroxybutanoate, EH, ethyl hexanoate, MH, 6-methyl-5-hepten-2-one, BZ, benzaldehyde, MP, 4-methylphenol, PO, paraffin oil (solvent blank).

To identify new genes involved in olfactory neuron function we screened 482 lines carrying homozygous viable EMS-induced mutations on chromosome III (from the Zuker Collection [25]) for defects in olfactory responses. Electroantennograms (EAGs) were employed to measure voltage changes across the antennal epithelium in response to a set of odorants known to excite a variety of different ORN classes. We identified one line (*ll2*) in which homozygous mutant flies showed reduced EAG responses to most tested odorants compared to heterozygous controls (p<0.05, Fig. 1A). Neurons on the second olfactory organ, the maxillary palp, showed an equivalent reduction in response to odorants in the mutant line (p<0.05, Fig. 1B). In contrast to the general odorants, the EAG response to carbon dioxide, which is generated by one specific antennal ORN class (ab1C) and mediated by two gustatory receptor genes, was unaffected in the mutant (Fig. 1A).

We next used genomic deficiencies to map the mutation. We found that flies trans-heterozygous for the *ll2* chromosome and either *Df(3R)Exel7312* or *Df(3R)Exel8155* showed the same mutant phenotype as homozygous *ll2* flies (Fig. 1C). This localised the mutation to an 86.5kb genomic region that contains 14 annotated genes (Fig. 1D). Whole genome sequencing experiments identified a nonsense mutation in the *CG14741* gene within this region (genomic location 7,902,447, mutation C7902447T). *CG14741* has seven predicted isoforms; the nonsense mutation is in the first coding exon common to all predicted isoforms and thus is predicted to severely truncate all proteins encoded by the locus. The *CG14741* gene has not previously been functionally characterised. However, sequence comparisons reveal it belongs to the type 4 P-type ATPase family of integral membrane transporter proteins [26]. This group of proteins includes four human members named ATPB1-4 and *CG14741* as the sole *Drosophila* representative. Henceforth we refer to the gene *CG14741* as *dATP8B*.

To confirm that the nonsense mutation in *dATP8B* was the cause of the olfactory defects observed in the *ll2* line we examined an independent mutant allele, a line containing a *piggyBac* element inserted in the coding region and predicted to affect all the isoforms (*dATP8B ^f05203^*, Fig. 1D). Flies homozygous for the *dATP8B ^f05203^* allele showed the same EAG defect as homozygotes for the original EMS allele (p<0.05, Fig. 1C). This phenotype was reverted when the *piggyBac* insertion was precisely excised (Fig. 1E), confirming that the olfactory defect is due to the insertion in *dATP8B*. We also showed that the EMS and *piggyBac* insertion alleles failed to complement; trans-heterozygotes for the two mutant alleles also exhibited the olfactory defect (p<0.05, Fig. 1C). Together, these data confirm that mutations in *dATP8B* cause a severe reduction in ORN responses.

**Figure 2:**
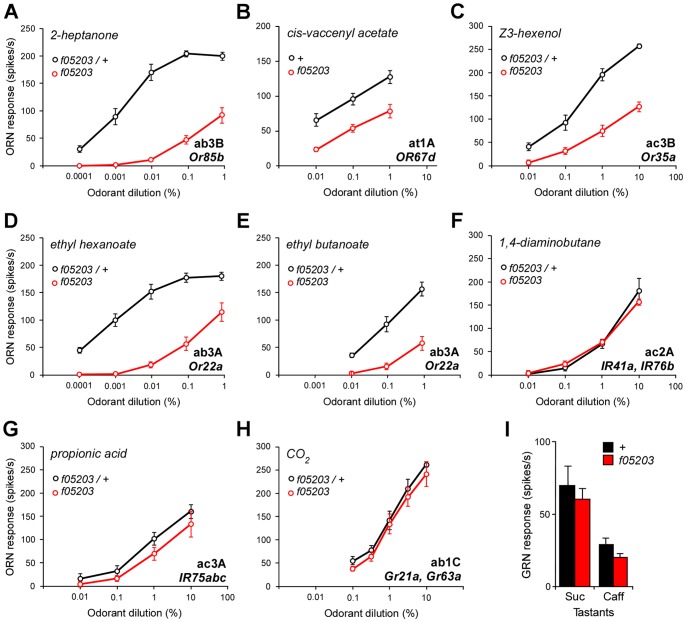
*dATP8B* is required for responses of *Or* but not *IR* or *Gr-* expressing sensory neurons. (A–C) Mutations in *dATP8B* reduce the sensitivity of *Or*-expressing neurons to their major ligands. Dose-response curves for neurons located in three different morphological types of sensilla are shown; (A) ab3B neurons in basiconic sensilla (*Or85b*) to 2-heptanone, (B) at1A neurons in trichoid sensilla (*Or67d*) to cis-vaccenyl acetate, (C) ac3B neurons in coeloconic sensilla (*Or35a*) to Z3-hexenol. In all cases sensitivity is significantly lowered for all doses in homozygous *dATP8B^f05203^* flies (red) compared to controls (black, t-test, Bonferroni, n = 6–9, recorded from 4–8 flies). (D–E) Mutations in *dATP8B* differentially alter responses of ab3A neurons (*Or22a*) to a major ligand ethyl hexanoate (D) and a minor ligand ethyl butanoate (E). (n = 7 sensilla, recorded from 5–6 flies). (F–I) Neurons expressing *IR* or *Gr* receptors are not affected by *dATP8B* mutations. Four different types of neurons are shown; (F) responses of ac2A neurons in coeloconic sensilla (*IR41a* and *IR76b*) to 1,4-diaminobutane, (G) responses of ac3A neurons in coeloconic sensilla (*IR75a,b and c*) to propionic acid, (H) responses of ab1C neurons in basiconic sensilla (*Grs21a* and *63a*) to CO_2_, (I) responses from single neurons in labellar taste sensilla expressing *Gr* genes to 100 mM sucrose (Suc) or 10 mM caffeine (Caff) (mean ± SEM). In all cases control and mutant responses are not significantly different (n = 6–10 sensilla from 3–5 flies, t-tests, Bonferroni). Controls are either wild type or heterozygous *dATP8B^f05203^* mutants.

In our initial EAG recordings we noted that the *dATP8B^ll2^* mutant flies had normal responses to carbon dioxide (CO_2_). Unlike the other odorants tested, which are detected by ORNs that express members of the *Or* receptor family, CO_2_ is detected by the ab1C ORN which expresses two members of the *Gr* receptor family, *Gr21a* and *Gr63a* [21], [22]. These data suggested that the mutation might be specifically affecting *Or*-expressing ORNs rather than all ORNs. To determine if this was the case we characterised mutant responses from selected ORN classes on the antenna whose responses are determined by members of all three different receptor gene families. In each neuron type we examined that expresses *Or* genes we found that the responses to ligands were substantially reduced over a range of concentrations (Fig. 2). This was the case regardless of the morphological type of sensillum, as we found greatly reduced responses to 2-heptanone from the ab3B neuron in basiconic sensilla (Fig. 2A), to cis-vaccenyl acetate from the at1A neuron in trichoid sensilla (Fig. 2B), and to Z3-hexenol from the ac3B neuron in coeloconic sensilla (Fig. 2C).

These experiments also confirmed a finding we had noted from our EAG recordings, namely that the responses of *dATP8B* mutant flies, while greatly reduced, are not completely abolished. We found that at high odorant concentrations mutant neurons were still able to fire at relatively high rates (an average of 100 spikes per second; Figs. 2A–C). We also noted that the effect of the mutation can be different for different odorants activating the same receptor. For the ab3A neuron, which expresses *Or22a*, we found that the sensitivity to its high affinity ligand ethyl hexanoate was reduced by three log steps (Fig. 2D). However, when we recorded responses from ab3A neurons for a lower affinity ligand, ethyl butanoate, we found that the curve is shifted much less (Fig. 2E). This suggests that sensitivity to different ligands of the Or22a receptor is affected by the *dATP8B* mutation to differing extents.

In contrast, we found that mutations in *dATP8B* have no effect on responses of ORNs that express *IR* or *Gr* genes. This was the case for two ORN types that express *IR* genes; the responses to 1,4-diaminobutane of the ac2A neuron (Fig. 2F) and to propionic acid of the ac3A neuron (Fig. 2G) were unaffected in the mutant. Consistent with the initial EAG data, in *dATP8B* mutants the CO_2_ response from the ab1C neuron, which is determined by *Gr* genes, is not significantly reduced for any of the concentrations that evoke a wide range of excitation levels (Fig. 2H). We also performed recordings from gustatory neurons on the labellum expressing members of the *Gr* family, and showed that in *dATP8B* mutants the response to both sucrose and caffeine, mediated by different Gr receptors [23], was unaffected (Fig. 2I). Taken together, these data strongly suggest that *dATP8B* is specifically required for the function of *Or*-expressing neurons, and not for chemosensory neurons in general.

**Figure 3:**
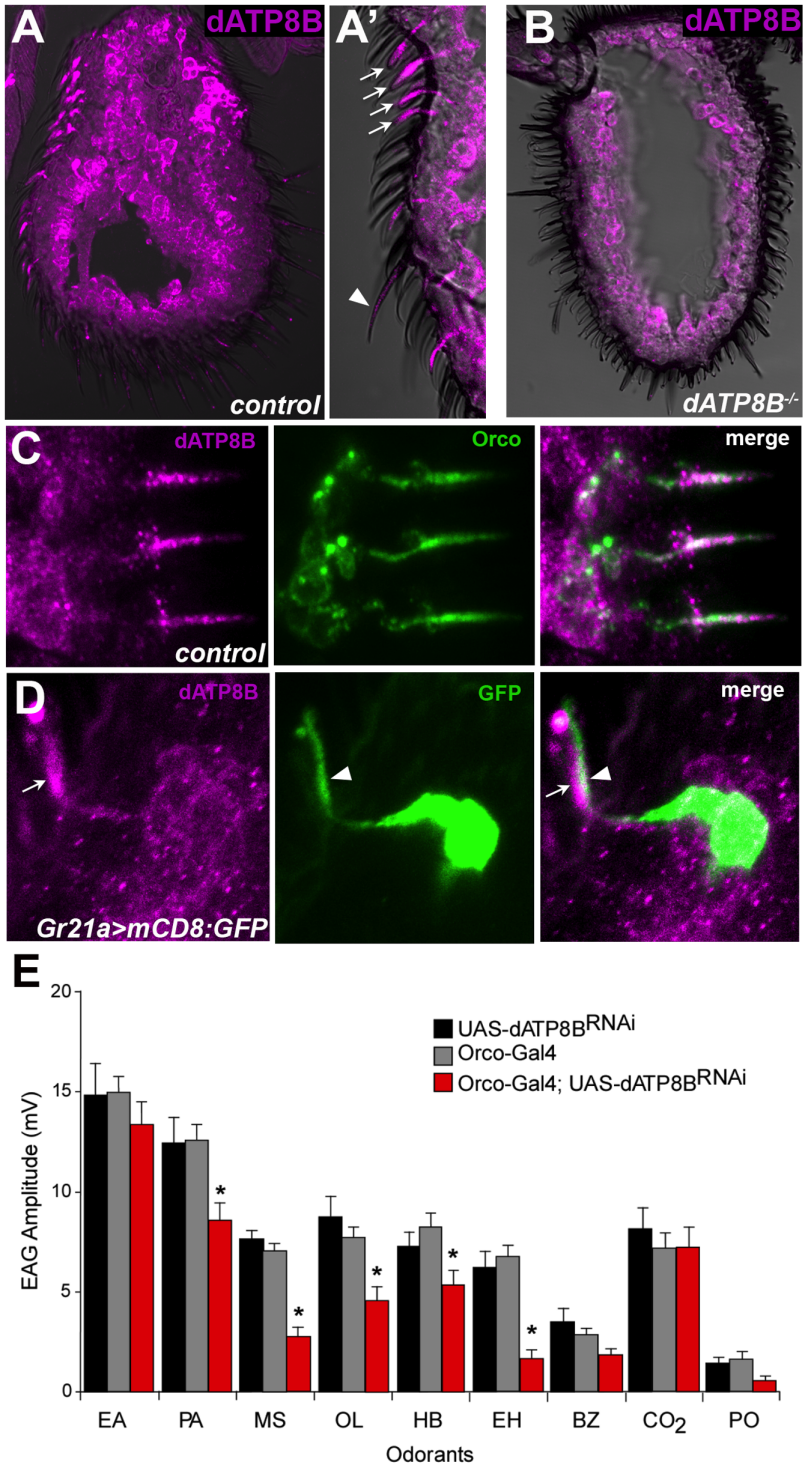
*dATP8B* is expressed and required in *Or-*expressing olfactory receptor neurons. (A–A′) dATP8B protein localises to the dendrites of ORNs. 14 µm thick antennal sections from wild type flies (CS-5) were stained for anti-dATP8B (magenta). Strong anti-dATP8B staining is seen in the shafts of the sensilla (the location of the outer dendrites) of basiconic (arrows) and trichoid (arrowhead) sensilla. Staining is also seen in the inner dendrites and cell bodies, however this staining is also present in *dATP8B* mutants. (B) The strong outer dendrite staining of anti-dATP8B is absent in *dATP8B* mutants (*ll2*/*Df(3R)Exel8155*), indicating it is specific for dATP8B. (C) dATP8B and Orco co-localise in the outer dendrites. 14 µm thick antennal sections from wild type flies were stained for anti-dATP8B (magenta) and Orco (green). (D) dATP8B is absent from the outer dendrites of *Gr21a*-expressing (ab1C) neurons. 14 µm thick antennal sections from Gr21a>mCD8:GFP flies were stained for anti-dATP8B (magenta) and anti-GFP (green). (E) In *Orco-GAL4: UAS-dATP8B^RNAi^* flies (red bars) the EAG response is significantly reduced compared to controls (black and grey bars, t-test, Bonferroni, *p<0.05*) for some of the same odorants that are affected by the two *dATP8B* mutant alleles. Bars represent mean EAG responses ± SEM (n = 6–10). Odorants are: EA, ethyl acetate, PA, pentyl acetate, MS, methyl salicylate, OL, 1-octen-3-ol, HB, ethyl 3-hydroxybutanoate, EH, ethyl hexanoate, BZ, benzaldehyde, PO, paraffin oil (solvent blank).

dATP8B was identified in a previous study as being present in the antennal proteome [27]. To confirm its expression in antennae and to determine the cell type in which dATP8B is expressed we performed immunohistochemistry using an anti-dATP8B antibody. Strong staining was seen within the shafts of the sensilla, the location of the outer dendrites of the ORNs (Fig. 3A). Staining was observed in both basiconic and trichoid sensilla, which both house neurons expressing *Or* genes. We could not easily visualise the sensilla shafts of the coeloconic sensilla. Staining was absent from the outer dendrites in the sensilla shafts in mutants for *dATP8B* (Fig. 3B). Signal was also observed in the inner dendrites and the cell bodies, however this signal was also observed in mutants for *dATP8B*, albeit more weakly, (compare Figs. 3A and B), and thus may be largely background staining.

Given *dATP8B* function seems to only be required in the *Or*-expressing neurons we next asked if it is specifically expressed in *Or*-expressing, but not *IR* or *Gr-*expressing, neurons. We confirmed that dATP8B is expressed in the *Or*-expressing neurons by showing that it co-localises with Orco (Fig. 3C). We then asked if dATP8B is expressed in the ab1C neurons that express *Gr21a*. These neurons are housed in ab1 basiconic sensilla together with three other neurons, which all express *Or* genes. We visualised the ab1C neurons by using *Gr21a-Gal4* to drive the membrane-localised mCD8:GFP and staining with anti-GFP. Strong GFP signal was observed in the outer and inner dendrites and cell bodies (Fig. 3D). Double staining with anti-GFP and anti-dATP8B showed that dATP8B is absent from the outer dendrites of *Gr21a*-expressing (ab1C) neurons (Fig. 3D). We saw many examples where the GFP-positive dendrites of the ab1C neurons ran parallel to but did not overlap dATP8B-positive dendrites within the sensilla shafts. We conclude that dATP8B localises to the outer dendrites in *Or*-expressing neurons, and likely does not in *Gr*-expressing neurons.

We also showed that *dATP8B* function is required in the ORNs, rather than another cell type, by using RNA interference (RNAi) to knock down *dATP8B* in the *Or*-expressing ORNs using an *Orco* driver. In *Orco-GAL4:UAS-dATP8B^RNAi^* flies the EAG response was significantly reduced for some of the odorants for which we also saw reduced responses in the two *dATP8B* mutant alleles, for example methyl salicylate and ethyl hexanoate (Fig. 3E). Although this defect is much less severe than seen in the loss of function mutants (Fig. 1A), taken together with the immunohistochemistry data these results suggest that *dATP8B* has a functional role in ORNs, rather than in another cell type such as support cells. As Orco has a relatively late onset of expression in pupal development [5], this result also suggests that *dATP8B* plays a role in these neurons after the initial development of olfactory sensilla.

**Figure 4:**
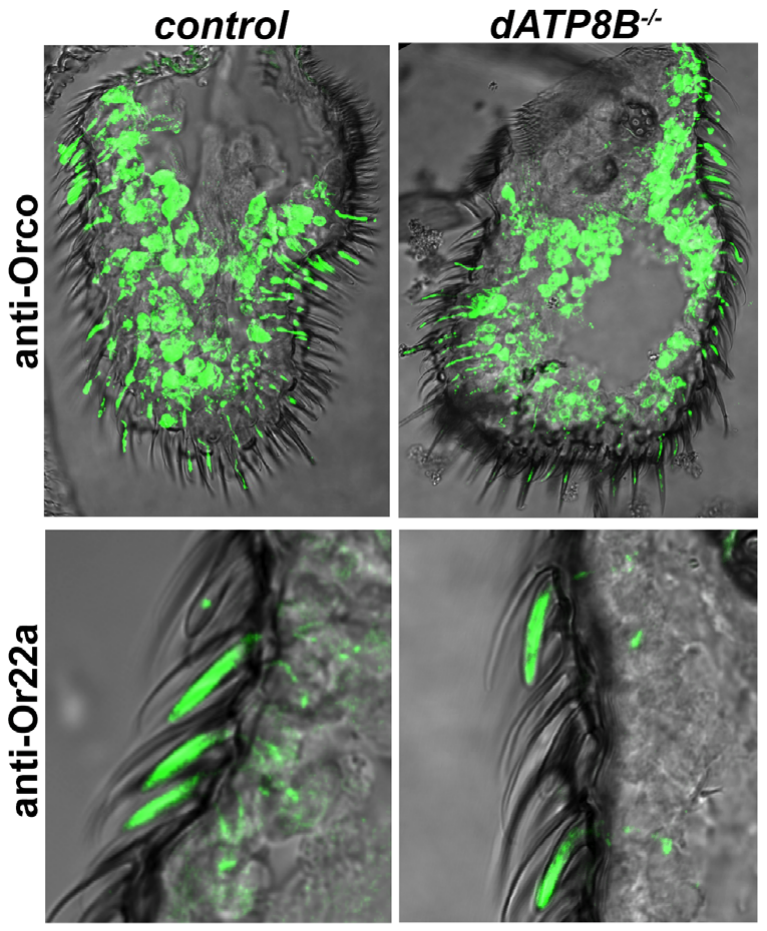
Orco and Or22a localize normally to the dendrites in *dATP8B* mutants. 14 µm thick antennal sections from wild type flies (CS-5) were stained for anti-Orco or for anti-Or22a. No difference in either Orco or Or22a localisation to the outer dendrites was observed in *dATP8B* mutants (*dATP8B^f05203^*) compared to control flies.

The phenotype of *dATP8B* mutants bears a strong resemblance to that of mutations in the *Orco* gene, which is required for the localisation and function of the Or receptors, but not for the IR or Gr receptors [5], [15], [16]. We thus asked if loss of *dATP8B* caused Orco itself and/or the other Or proteins to be incorrectly localized. We tested this by using antibodies to examine the localization of both Orco and Or22a. In wild type flies anti-Orco staining is seen in both the outer dendrites and the cell bodies, for anti-Or22a there is strong staining in the outer dendrites only (Fig. 4). No difference in localization of either Orco or Or22a was observed in *dATP8B* mutants (Fig. 4). In addition, no noticeable difference was observed in the length or shape of the dendrites in the mutant. This confirmed an initial finding that the overall appearance of the olfactory sensilla in the mutant is normal (Fig. S1).

## Discussion

dATP8B is a member of the type 4 subfamily (P4-ATPases) of P-type ATPases. Unlike the other subfamilies, most of which encode ion transporters, the P4-ATPases are believed to share a distinct function as phospholipid translocases or “flippases” [26]. Eukaryotic plasma membranes have an asymmetrical distribution of phospholipids across the bilayer, with sphingolipids and phosphatidylcholine enriched in the exoplasmic leaflet, and more polar lipids such as phosphatidylserine and phosphatidylethanolamine enriched in the cytoplasmic leaflet. Phospholipid flippases contribute to asymmetry by “flipping” phospholipids from the exoplasmic to the cytoplasmic leaflet [28]. The physiological significance of this asymmetry is not well understood but it seems to be important for critical membrane processes such as vesicle trafficking and intracellular signalling [26], [29]. Disruption of the asymmetry may affect the conformation, membrane insertion, or trafficking, of integral membrane proteins. Alternatively it could affect lipid-signalling molecules, or membrane fluidity or bending. There are six members of the phospholipid flippase family in *Drosophila* and *C.elegans* and 14 in humans [26]. Genetic studies in *C.elegans* have suggested that the different members have different functions [30]. One of the six *Drosophila* flippases (CG33298) has been implicated in secretory vesicle formation and cholesterol homeostasis [31], the others have been uncharacterized to date.

dATP8B is the *Drosophila* homologue of four mammalian ATP8B proteins [26]. Of these only ATP8B1 has been substantially studied. Mutations in human ATP8B1 are associated with intrahepatic cholestasis [32] and also cause hearing loss [33]. The protein localizes to the apical plasma membrane of hepatocytes [32], [34], where it is thought to play a role in protection from the detergent effects of bile salts [35]. In hair cells, the sensory cells of the inner ear, ATP8B1 has been shown to localize to the stereocilia that transduce the mechanical vibrations in the cochlea [33]. ATP8B1 deficient mice exhibit a progressive degeneration of cochlea hair cells, possibly due to changes in the mechanical properties of stereocilia or the disruption of a Ca^2+^ transporter crucial for sensory transduction [33].

Here we have found that *dATP8B* is essential specifically for the responses of *Or*-expressing neurons, and not for *IR* or *Gr*-expressing neurons in general. Our expression studies suggest that *dATP8B* is not expressed in the latter, although further studies are needed to confirm the generality of this. Several lines of evidence suggest that mutation in *dATP8B* is not disrupting the development or morphology of the Or-expressing neurons and that its function is required in adult ORNs. First, in spite of the dramatic effects on sensitivity to odorants, the affected ORNs are still functional neurons, as evidenced by some response at high odorant doses, and have normal morphological appearance. Second, we found two examples where *Gr* or *IR*-expressing unaffected neurons (ac3A and ab1C) are housed in the same sensilla as affected *Or*-expressing neurons. This indicates that the defect is intrinsic to the *Or*-expressing neurons, and is not due to altered sensillum morphology or perireceptor processes.

Our data suggest that *dATP8B* is not required for the localization of the Or proteins to the dendrites, although we note that we have only examined Orco and one ligand-binding Or protein (Or22a) and thus cannot completely rule out effects on localisation of other Or proteins. Nonetheless, it seems most likely that dATP8B is necessary instead for the function of the Or receptor complex and for primary receptor signal transduction processes. This is further supported by our finding that *dATP8B* mutations differentially affect sensitivity of an individual receptor to different odorants. At present there is no biochemical evidence that dATP8B functions as a phospholipid flippase, however all members of this family of P4-ATPases for which biochemical assays have been performed do function to translocate phospholipids [26], [28], [29]. If dATP8B functions as expected there are several ways it could affect Or signal transduction. An altered phospholipid composition of the plasma membrane in *dATP8B* mutants may affect Or-Orco interactions, or interfere with binding of odorants to the receptor complex. Alternatively, reduced availability or activity of membrane-localised signalling molecules may affect Or signalling. For example, phosphatidylserine (PS) is required for activation of PKC once it translocates to the plasma membrane in response to increases in diacylglycerol [36]. In *dATP8B* mutants reduced availability of PS in the inner leaflet could thus lead to loss of PKC activation. PKC has functions in many signalling pathways, and has been suggested to be important for Orco activation [8]. Another possibility is that mutations in *dATP8B* affect the minor plasma membrane phospholipid phosphatidylinositol 4,5-bisphosphate (PIP_2_). PIP_2_ is found primarily in the cytoplasmic leaflet of the membrane and does not flip between leaflets, but levels could potentially be disrupted by flippase disfunction. PIP_2_ has many signalling roles. Its cleavage products inositol 1,4,5-trisphosphate and diacylglycerol are key components of G protein-activated signalling pathways. In addition, a number of families of ion channel and ion transporter proteins, for example the transient receptor potential channels, are dependent on PIP_2_ for their activation [37].

In conclusion, we have identified a new olfactory gene, *dATP8B*, which is specifically required for odour responses of *Or*-expressing, but not *Gr* or *IR*-expressing, sensory neurons in *Drosophila*. Given the very high level of homology of dATP8B to known phospholipid flippases, our findings suggest a specific role for cell membrane phospholipids in Or receptor signalling, as well as providing further evidence for fundamental differences between the signalling mechanisms of the different families of insect olfactory receptors. Further studies of this interesting gene may provide insight into the potentially complex mechanisms of Or signalling.

## Materials and Methods

### *Drosophila* stocks

*Drosophila* stocks were reared on yeasted semolina/syrup medium in 30 ml vials at 22°C under a natural daylight cycle. All crosses were performed at 22°C. Flies carrying the *dATP8B^ll2^* mutation were part of a collection of mutagenized stocks obtained from Charles Zuker's laboratory [25]. The *piggyBac* insertion line *dATP8B^f05203^* (BL18847) and deficiency lines *Df(3R)Exel7312* (BL7966) and *Df(3R)Exel8155* (BL7967) were obtained from Bloomington Stock Center. The RNAi line for *dATP8B* was obtained from the Vienna *Drosophila* RNAi Center (v102648). The *Orco*-Gal4 line was obtained from Leslie Vosshall (Rockefeller University) and the *Gr21a*-Gal4 line from Kristin Scott at University of California Berkeley.

### Electrophysiological recordings and data analysis

#### Recordings from whole olfactory organs

We recorded electrical signals from whole antennae (electroantennogram, EAG) and maxillary palps (electropalpogram, EPG) as described in Tom et al. 2011 [38]. A single fly was immobilized and a reference electrode inserted in the eye. For EAGs the recording electrode was placed on the surface of the antenna and for EPGs on the palp. Changes in voltage (mV) in response to 1 s stimulation with odorants were amplified using an active probe and a serial-IDAC amplifier (Syntech, Hilversum, the Netherlands). EAGs and EPGs represent the summed activity of a population of ORNs.

#### Recordings from single olfactory sensilla

Activity of individual olfactory receptor neurons was studied using the single sensillum recording (SSR) technique as described elsewhere [39]. Action potentials were recorded by a glass electrode inserted at the base of an olfactory sensillum and amplified via an IDAC-4 amplifier (Syntech). Action potential firing rates during 500 ms stimulations were analysed by subtracting firing rates during the 2 seconds before stimulation. Action potentials from the different neurons in a single sensillum were separated as in de Bruyne et al. [39]. A two-tailed Student's t-test with a Bonferroni correction for multiple comparisons was used to compare firing rates. Odor stimulation for SSR, EAG and EPG recordings was by injecting volatiles from 5 ml disposable syringes into an airstream blown over the preparation. All odorants were at highest available purity (>98%, Sigma-Aldrich) and dissolved in paraffin oil at different dilution from 0.0001 to 10% v/v. Because of its low volatility, the *Drosophila* pheromone cis-vaccenyl acetate (>98%, Cayman Chemicals) was dissolved in hexane and delivered from a pasteur pipette that was briefly heated prior to use. Male flies of age 3–7 days were used for all electrophysiological recordings, except for the RNAi experiment where newly emerged male flies were incubated at 25°C for exactly 7 days before recordings were performed.

#### Recordings from taste sensilla

Single sensillum tip recordings were performed from large (L-type) and intermediate (Ib-type) sensilla in the labellum as described earlier [20]. Male flies were aged 3–7 days and prepared for recordings by insertion of a glass micropipette reference electrode filled with Ringer's solution. Tastants were dissolved in 30 mM tricholine citrate, which was used as the electrolyte for the recording electrode. Action potentials obtained by using a TasteProbe and IDAC-4 amplifier (Syntech), were counted during the 500-ms period after initial contact with the stimulus solution in the recording electrode, and multiplied by two to obtain firing rates in spikes per second. A two-tailed Student's t-test was used to compare firing rates between mutant and wild-type flies. Sucrose and caffeine were purchased from Sigma Aldrich.

### Genome sequencing and data analysis

Genomic DNA was extracted from adult heterozygous males using a QIAGEN Genomic-tip 20/G. A paired-end library with ∼300 bp insert size was prepared and sequenced by the Australian Genome Research Facility. In total ∼19 million 100 bp paired-end reads were generated using 0.5 lane on the Illumina HiSeq system. Sequencing reads were mapped to the *Drosophila* reference genome (Release 5 assembly) using BWA (Version 0.5.9) with default settings [40]. Integrative Genomics Viewer (Version 1.5.64) was used to visually inspect overall mapping quality of the candidate region [41]. After quality validation, consensus was generated using SAMtools (Version 0.1.13) with default settings [42]. Sequence variations were annotated using ANNOVAR's gene-based annotation option [43] with FlyBase Release 5.36 annotation [44]. SNPs from the *Drosophila melanogaster* Genetic Reference Panel [45] were used to filter naturally occurring variations. All computations were performed on the Monash Sun Grid. The nonsense mutation in *dATP8B* was verified with Sanger Sequencing using an independently prepared genomic DNA sample. The following primers were used to amplify a 655 bp region flanking the mutation site: forward primer 5′CATACGCATCCTTAACAGCC3′, reverse primer 5′ACCCAACAAATCCGATGACC3′.

### Antibody production

cDNAs encoding six different regions of the CG14741-PB isoform (PE1, a.a. 2–236; PE2, 261–450; PE3, 527–630; PE4, 655–1115; PE5, 967–1359; PE6, 1562–1726) that are not part of predicted transmembrane domains were cloned into a pET100/D-TOPO vector (Invitrogen, Carlsbad, CA) such that the expressed peptide was tagged N-terminally with 6×His. The 6×His:PE1-6 peptides were expressed in *E. coli* and purified using a Ni-NTA column (Invitrogen). Peptides PE3, 5, and 6 were soluble and were individually injected into two guinea pigs by Cocalico Biologicals (Reamstown, PA). The obtained antisera (90 day protocol) were screened for antibodies against dATP8B using Western blots of both fly head protein extracts and the bacterially expressed peptides. The antisera obtained from injecting PE5 and PE6 showed positive signals and PE6 was used for immunostaining.

### Immunohistochemistry

Antibodies and dilutions used were as follows: guinea pig anti-dATP8B (1∶10,000); rabbit anti-Orco (1∶5,000; Vosshall lab); rabbit anti-Or22a (1∶1,000; Vosshall lab); rabbit anti-GFP (1∶1,000; Life Technologies). Secondary antibodies raised in mouse, rabbit and guinea pig were Alexa-conjugated (Alexa Fluro 488 at 1∶250–500, Alexa Fluro 568 at 1∶500) (Molecular Probes). 14 µM cryo-sectioned adult heads were mounted on SuperFrost Plus slides (Thermo Scientific), dried for up to 3 hours, and then fixed in 4% paraformaldehyde/PBS for 30 mins at room temperature. Samples were washed for 10 minutes three times with PBST (PBS, 0.3% Triton-X-100), incubated in Block (5% normal goat serum in PBST) for 2 hours at room temperature and then incubated with the primary antibodies diluted in Block overnight at 4°C. After three 10 minutes washes with PBST, samples were incubated with secondary antibodies diluted in Block for 2 hours at room temperature. Sections were washed for 10 minutes three times with PBST before being mounted in Vectashield (Vector Labs). Samples were viewed and images acquired using a Nikon C1 confocal microscope.

## Supporting Information

Figure S1 The olfactory sensilla of the antenna show normal morphology in *dATP8B* mutants. Scanning electron micrographs of third antennal segments and maxillary palps from wild type and homozygous *dATP8B ^ll2^* mutants. No obvious abnormalities are seen in the mutant flies. Scale bars are 20 µm.
(TIF)

## References

[1] Benton R, Sachse S, Michnick SW, Vosshall LB (2006) Atypical membrane topology and heteromeric function of *Drosophila* odorant receptors in vivo. PLoS Biol 4 (2) e20. 16402857 10.1371/journal.pbio.0040020 PMC1334387

[2] Smart R, Kiely A, Beale M, Vargas E, Carraher C, et al (2008) *Drosophila* odorant receptors are novel seven transmembrane domain proteins that can signal independently of heterotrimeric G proteins. Insect Biochem Mol Biol 38: 770–780. 18625400 10.1016/j.ibmb.2008.05.002

[3] Sato K, Pellegrino M, Nakagawa T, Nakagawa T, Vosshall LB, et al (2008) Insect olfactory receptors are heteromeric ligand-gated ion channels. Nature 452: 1002–1006. 18408712 10.1038/nature06850

[4] Wicher D, Schäfer R, Bauernfeind R, Stensmyr MC, Heller R, et al (2008) *Drosophila* odorant receptors are both ligand-gated and cyclic-nucleotide-activated cation channels. Nature 452: 1007–1011. 18408711 10.1038/nature06861

[5] Larsson MC, Domingos AI, Jones WD, Chiappe E, Amrein H, et al (2004) Or83b encodes a broadly expressed odorant receptor essential for *Drosophila* olfaction. Neuron 43: 703–714. 15339651 10.1016/j.neuron.2004.08.019

[6] Jones PL, Pask GM, Rinker DC, Zwiebel LJ (2011) Functional agonism of insect odorant receptor ion channels. Proc Natl Acad Sci USA 108: 8821–8825. 21555561 10.1073/pnas.1102425108 PMC3102409

[7] Nakagawa T, Pellegrino M, Sato K, Vosshall LB, Touhara K (2012) Amino acid residues contributing to function of the heteromeric insect olfactory receptor complex. PLoS ONE 7: e32372. 22403649 10.1371/journal.pone.0032372 PMC3293798

[8] Stengl M, Funk NW (2013) The role of the coreceptor Orco in insect olfactory transduction. J Comp Physiol A 199: 897–909. 10.1007/s00359-013-0837-3 23824225

[9] Sargsyan V, Getahun MN, Llanos SL, Olsson SB, Hansson BS, et al (2011) Phosphorylation via PKC regulates the function of the *Drosophila* odorant co-receptor. Front Cell Neurosci 5: 5. 21720521 10.3389/fncel.2011.00005 PMC3118453

[10] Kain P, Chakraborty TS, Sundaram S, Siddiqi O, Rodrigues V, et al (2008) Reduced odor responses from antennal neurons of Gqα, phospholipase Cβ, and rdgA mutants in *Drosophila* support a role for a phospholipid intermediate in insect olfactory transduction. J Neurosci 28: 4745–55. 18448651 10.1523/JNEUROSCI.5306-07.2008 PMC3844817

[11] Yao CA, Carlson JR (2010) Role of G-proteins in odor-sensing and CO_2_-sensing neurons in *Drosophila* . J Neurosci 30: 4562–72. 20357107 10.1523/JNEUROSCI.6357-09.2010 PMC2858456

[12] Deng Y, Zhang W, Farhat K, Oberland S, Gisselmann G, et al (2011) The stimulatory Gαs protein is involved in olfactory signal transduction in *Drosophila* . PLoS One 6 (4) e18605. 21490930 10.1371/journal.pone.0018605 PMC3072409

[13] Getahun MN, Olsson SB, Lavista-Llanos S, Hansson BS, Wicher D (2013) Insect odorant response sensitivity is tuned by metabotropically autoregulated olfactory receptors. PLoS One 8 (3) e58889. 23554952 10.1371/journal.pone.0058889 PMC3595248

[14] Benton R, Vannice KS, Gomez-Diaz C, Vosshall LB (2009) Variant ionotropic glutamate receptors as chemosensory receptors in *Drosophila* . Cell 136: 149–162. 19135896 10.1016/j.cell.2008.12.001 PMC2709536

[15] Abuin L, Bargeton B, Ulbrich MH, Isacoff EY, Kellenberger S, et al (2011) Functional architecture of olfactory ionotropic glutamate receptors. Neuron 69: 44–60. 21220098 10.1016/j.neuron.2010.11.042 PMC3050028

[16] Ai M, Blais S, Park J-Y, Min S, Neubert TA, et al (2011) Ionotropic glutamate receptors IR64a and IR8a form a functional odorant receptor complex *in vivo* in *Drosophila* . J Neurosci 33: 10741–10749. 23804096 10.1523/JNEUROSCI.5419-12.2013 PMC3693055

[17] Clyne PJ, Warr CG, Carlson JR (2000) Candidate taste receptors in *Drosophila* . Science 287: 1830–1834. 10710312 10.1126/science.287.5459.1830

[18] Robertson HM, Warr CG, Carlson JR (2003) Molecular evolution of the insect chemoreceptor gene superfamily in *Drosophila melanogaster* . Proc Natl Acad Sci USA 100 (Suppl 2): 14537–14542. 14608037 10.1073/pnas.2335847100 PMC304115

[19] Hallem EA, Carlson JR (2006) Coding of odors by a receptor repertoire. Cell 125: 143–160. 16615896 10.1016/j.cell.2006.01.050

[20] Silbering AF, Rytz R, Grosjean Y, Abuin L, Ramdya P, et al (2011) Complementary function and integrated wiring of the evolutionarily distinct *Drosophila* olfactory subsystems. J Neurosci 31: 13357–13375. 21940430 10.1523/JNEUROSCI.2360-11.2011 PMC6623294

[21] Jones WD, Cayirlioglu P, Kadow IG, Vosshall LB (2007) Two chemosensory receptors together mediate carbon dioxide detection in *Drosophila* . Nature 445: 86–90. 17167414 10.1038/nature05466

[22] Kwon JY, Dahanukar A, Weiss LA, Carlson JR (2007) The molecular basis of CO2 reception in *Drosophila* . Proc Natl Acad Sci USA 104: 3574–3578. 17360684 10.1073/pnas.0700079104 PMC1805529

[23] Montell C (2009) A taste of the *Drosophila* gustatory receptors. Curr Opin Neurobiol 19: 345–353. 19660932 10.1016/j.conb.2009.07.001 PMC2747619

[24] Weiss LA, Dahanukar A, Kwon JY, Banerjee D, Carlson JR (2011) The molecular and cellular basis of bitter taste in *Drosophila* . Neuron 69: 258–272. 21262465 10.1016/j.neuron.2011.01.001 PMC3033050

[25] Koundakjian EJ, Cowan DM, Hardy RW, Becker AH (2004) The Zuker collection: a resource for the analysis of autosomal gene function in *Drosophila melanogaster* . Genetics 167: 203–206. 15166147 10.1534/genetics.167.1.203 PMC1470872

[26] Tanaka K, Fujimura-Kamada K, Yamamoto T (2011) Functions of phospholipid flippases. J Biochem 149: 131–143. 21134888 10.1093/jb/mvq140

[27] Anholt RRH, Williams TI (2010) The soluble proteome of the *Drosophila* antenna. Chem Senses 35: 21–30. 19917591 10.1093/chemse/bjp073 PMC2795394

[28] Van der Velden LM, Van de Graaf SFJ, Klomp LWJ (2010) Biochemical and cellular functions of P4 ATPases. Biochem J 431: 1–11. 20836764 10.1042/BJ20100644

[29] Paulusma CC, Oude Elferink RPJ (2010) P4 ATPases – The physiological relevance of lipid flipping transporters. FEBS Letters 584: 2708–2716. 20450914 10.1016/j.febslet.2010.04.071

[30] Lyssenko NN, Miteva Y, Gilroy S, Hanna-Rose W, Schlegel RA (2008) An unexpectedly high degree of specialization and a widespread involvement in sterol metabolism among the *C. elegans* putative aminophospholipid translocases. BMC Dev Biol 8: 96. 18831765 10.1186/1471-213X-8-96 PMC2572054

[31] Ma Z, Liu Z, Huang X (2012) Membrane phospholipid asymmetry counters the adverse effects of sterol overloading in the Golgi membrane of *Drosophila* . Genetics 190: 1299–1308. 22234859 10.1534/genetics.111.137687 PMC3316644

[32] Ujhazy P, Ortiz D, Misra S, Li S, Moseley J, et al (2001) Familial intrahepatic cholestasis 1: studies of localization and function. Hepatology 34: 768–775. 11584374 10.1053/jhep.2001.27663

[33] Stapelbroek JM, Peters TA, van Beurden DHA, Curfs JHAJ, Joosten A, et al (2009) ATP8B1 is essential for maintaining normal hearing. Proc Natl Acad Sci USA 106: 9709–9714. 19478059 10.1073/pnas.0807919106 PMC2700994

[34] Cai SY, Gautam S, Nguyen T, Soroka CJ, Rahner C, et al (2009) ATP8B1 deficiency disrupts the bile canalicular membrane bilayer structure in hepatocytes, but FXR expression and activity are maintained. Gastroenterology 136: 1060–1069. 19027009 10.1053/j.gastro.2008.10.025 PMC3439851

[35] Folmer DE, van der Mark VA, Ho-Mok KS, Oude Elferink RPJ, Paulusma CC (2009) Differential effects of progressive familial intrahepatic cholestasis type 1 and benign recurrent intrahepatic cholestasis type 1 mutations on canalicular localization of ATP8B1. Hepatology 50: 1597–1605. 19731236 10.1002/hep.23158

[36] Orr JW, Newton AC (1992) Interaction of Protein Kinase C with Phosphatidylserine. 2. Specificity and Regulation. Biochemistry 31: 4667–73. 1581317 10.1021/bi00134a019

[37] Suh B-C, Hille B (2008) PIP_2_ is a necessary factor for ion channel function: How and why? Annu Rev Biophys 37: 175–95. 18573078 10.1146/annurev.biophys.37.032807.125859 PMC2692585

[38] Tom W, de Bruyne M, Haehnel M, Carlson JR, Ray A (2011) Disruption of olfactory receptor neuron patterning in Scutoid mutant *Drosophila* . Mol Cell Neurosci 46: 252–261. 20875862 10.1016/j.mcn.2010.09.008 PMC3019251

[39] de Bruyne M, Clyne PJ, Carlson JR (1999) Odor coding in a model olfactory organ: the *Drosophila* maxillary palp. J Neurosci 19: 4520–4532. 10341252 10.1523/JNEUROSCI.19-11-04520.1999 PMC6782632

[40] Li H, Durbin R (2009) Fast and accurate short read alignment with Burrows-Wheeler transform. Bioinformatics 25: 1754–1760. 19451168 10.1093/bioinformatics/btp324 PMC2705234

[41] Robinson JT, Thorvaldsdóttir H, Winckler W, Guttman M, Lander ES, et al (2011) Integrative genomics viewer. Nat Biotechnol 29: 24–26. 21221095 10.1038/nbt.1754 PMC3346182

[42] Li H, Handsaker B, Wysoker A, Fennell T, Ruan J, et al (2009) The Sequence Alignment/Map format and SAMtools. Bioinformatics 25: 2078–2079. 19505943 10.1093/bioinformatics/btp352 PMC2723002

[43] Wang K, Li M, Hakonarson H (2010) ANNOVAR: functional annotation of genetic variants from high-throughput sequencing data. Nucleic Acids Res 38: e164. 20601685 10.1093/nar/gkq603 PMC2938201

[44] Tweedie S, Ashburner M, Falls K, Leyland P, McQuilton P, et al (2009) FlyBase: enhancing *Drosophila* Gene Ontology annotations. Nucleic Acids Res 37: D555–9. 18948289 10.1093/nar/gkn788 PMC2686450

[45] Mackay TFC, Richards S, Stone EA, Barbadilla A, Ayroles JF, et al (2012) The *Drosophila melanogaster* Genetic Reference Panel. Nature 482: 173–178. 22318601 10.1038/nature10811 PMC3683990

