# Supplementary figures and images for: The Drosophila melanogaster Phospholipid Flippase dATP8B Is Required for Odorant Receptor Function
Source: PLoS Genet. 2014 Mar 20;10(3):e1004209. doi: 10.1371/journal.pgen.1004209 (PMC3961175; doi:10.1371/journal.pgen.1004209)

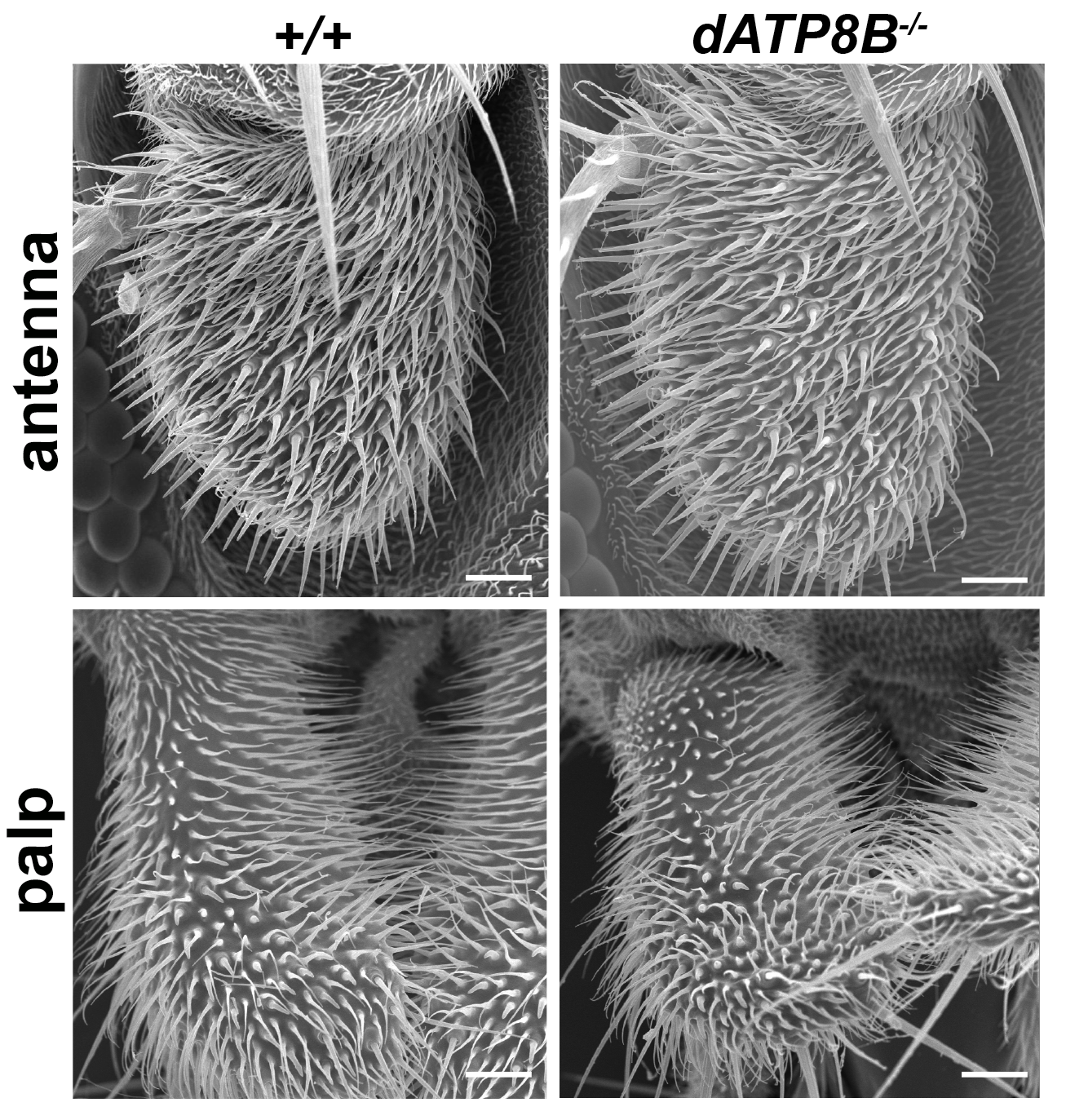

Supplement: Figure S1 — The olfactory sensilla of the antenna show normal morphology in dATP8B mutants. Scanning electron micrographs of third antennal segments and maxillary palps from wild type and homozygous dATP8B ll2 mutants. No obvious abnormalities are seen in the mutant flies. Scale bars are 20 µm. (TIF) [file pgen.1004209.s001.tif]
